# [Retracted] Effects of cisplatin on the proliferation, invasion and apoptosis of breast cancer cells following β-catenin silencing

**DOI:** 10.3892/ijmm.2024.5437

**Published:** 2024-10-07

**Authors:** Xidan Zhu, Jia Feng, Wenguang Fu, Xiaojia Shu, Xue Wan, Jinbo Liu

Int J Mol Med 45: 1838-1850, 2020; DOI: 10.3892/ijmm.2020.4543

Following the publication of this paper, it was drawn to the Editor's attention by a concerned reader that, for the cell migration and invasion assay data shown in Fig. 3A, C, E and G on p. 1843, a number of overlapping data sections were identified such that data which were intended to show the results from differently performed experiments had apparently been derived from the same original sources; moreover, these overlaps were featured in different alignments relative to their matching partners. In addition, other errors had been made during the process of compiling the figures; for example, the authors had overlooked indicating that the protein data shown in Fig. 1F were for β-catenin.

In view of the number of overlapping data panels that were identified in Fig. 3, the Editor of *International Journal of Molecular Medicine* has decided that this paper should be retracted from the Journal. The authors were asked for an explanation to account for these concerns, but the Editorial Office did not receive a satisfactory reply. The Editor apologizes to the readership for any inconvenience caused.

